# Efficient Carbon-Based CsPbBr_3_ Inorganic Perovskite Solar Cells by Using Cu-Phthalocyanine as Hole Transport Material

**DOI:** 10.1007/s40820-018-0187-3

**Published:** 2018-01-16

**Authors:** Zhiyong Liu, Bo Sun, Xingyue Liu, Jinghui Han, Haibo Ye, Tielin Shi, Zirong Tang, Guanglan Liao

**Affiliations:** 1 State Key Laboratory of Digital Manufacturing Equipment and Technology, Huazhong University of Science and Technology, Wuhan, 430074 People’s Republic of China; 2 Flexible Electronics Research Center, Huazhong University of Science and Technology, Wuhan, 430074 People’s Republic of China

**Keywords:** Perovskite solar cells (PSCs), Metal halide, CsPbBr_3_, Cu-phthalocyanine (CuPc), Carbon electrode

## Abstract

Metal halide perovskite solar cells (PSCs) have attracted extensive research interest for next-generation solution-processed photovoltaic devices because of their high solar-to-electric power conversion efficiency (PCE) and low fabrication cost. Although the world’s best PSC successfully achieves a considerable PCE of over 20% within a very limited timeframe after intensive efforts, the stability, high cost, and up-scaling of PSCs still remain issues. Recently, inorganic perovskite material, CsPbBr_3_, is emerging as a promising photo-sensitizer with excellent durability and thermal stability, but the efficiency is still embarrassing. In this work, we intend to address these issues by exploiting CsPbBr_3_ as light absorber, accompanied by using Cu-phthalocyanine (CuPc) as hole transport material (HTM) and carbon as counter electrode. The optimal device acquires a decent PCE of 6.21%, over 60% higher than those of the HTM-free devices. The systematic characterization and analysis reveal a more effective charge transfer process and a suppressed charge recombination in PSCs after introducing CuPc as hole transfer layer. More importantly, our devices exhibit an outstanding durability and a promising thermal stability, making it rather meaningful in future fabrication and application of PSCs.

**Electronic supplementary material:**

The online version of this article (10.1007/s40820-018-0187-3) contains supplementary material, which is available to authorized users.

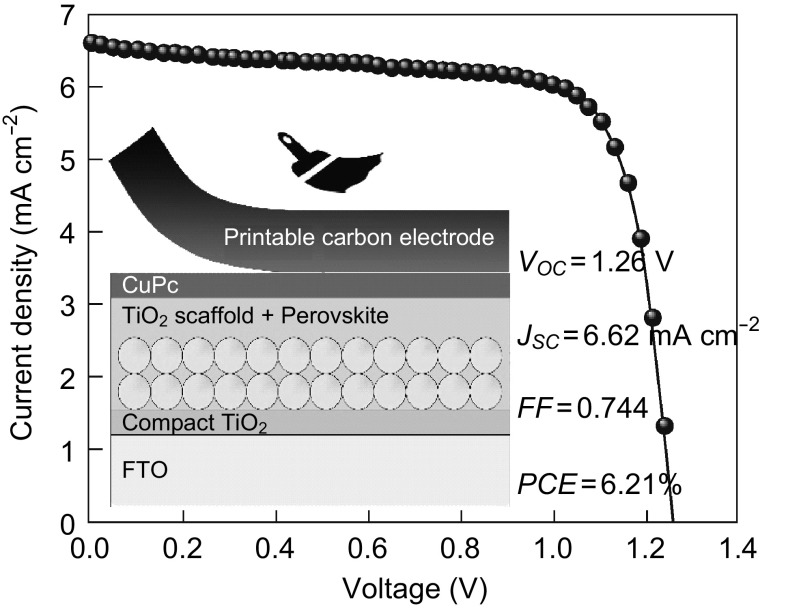

## Highlights

- Cu-phthalocyanine was employed as hole transport material for CsPbBr_3_ inorganic perovskite solar cells.
- The optimal device acquires a decent power conversion efficiency of 6.21%, over 60% higher than those of the hole transport material-free devices.
- The device exhibits an outstanding durability and a promising thermal stability.

## Introduction

Organic–inorganic perovskite solar cells (PSCs) are appearing as a hopeful new generation of photovoltaic technology and have revolutionized the prospects of emerging photovoltaic industry, because of the tremendous increase in device performance [1–6]. The outstanding photoelectric properties, such as high absorption coefficient, suitable and adjustable band gap [7–9], ambipolar charge transport [10–13], and long carrier diffusion length [14, 15], make perovskite materials very appropriate for light harvesting in photovoltaics. Since the breaking report from Miyasaka [16], power conversion efficiency (PCE) of such PSCs has reached a remarkable value (over 22%) in a short span [17–19], approaching the efficiency of commercialized c-Si solar cells and thin-film photovoltaic solar cells such as CdTe and Cu_2_ZnSn(Se,S)_4_ [20]. Despite the rapid increment in PCE associated with the evolution of new perovskite materials and novel fabrication techniques, the instability of PSCs remains unresolved. The mostly studied hybrid perovskite materials, for example methylammonium lead triiodide (MAPbI_3_) and formamidinium lead triiodide (FAPbI_3_), are forceless against moisture and heat. Some organic additives in commonly used HTMs, such as lithium bis(trifluoromethanesulfonyl)imide (LiTFSI) and *tert*-butylpyridine (tBP), are also hygroscopic and deliquescent, accelerating performance degradation [21–24]. Thus, precise environmental controls (gloveboxes or dryrooms) are often necessary during the fabrication of organic–inorganic hybrid PSCs. On the other side, efficient PSCs generally employ a p-type organic small-molecule or polymeric hole conductor, such as 2,2′,7,7′-tetrakis (*N*,*N*′-di-*p*-methoxyphenylamine)-9,9′-spirobifluorene (spiro-OMeTAD) [25], poly(3-hexylthiophene) (P3HT) [26], and poly(triarylamine) (PTAA) [27] as hole-extraction materials to boost device efficiencies. Discouragingly, these conventional HTMs suffer from disadvantages of high synthetic cost, thermal and chemical instability, and low hole mobility or low conductivity in their pristine form [28–30], seriously hindering the viable commercialization of the emerging PSC technology. The necessary doping techniques involved in improving their carrier density and conductivity further increase the cost in production. In addition, the high-energy-consuming coating process together with the consumption of noble metals as counter electrode (such as Au and Ag, widely used in efficient state-of-the-art PSCs) gives another problem for the commercialization of PSCs. To sum up, there are mainly three cruxes for the future up-scaling of PSCs: (1) exploring novel perovskite materials and HTMs with high stability against humidity and heat; (2) developing efficient, low-cost, durable, and scalable alternative HTMs that can replace currently used organic ones; (3) searching for low-cost and scalable substitutions for noble counter electrodes.

It has been proposed that inorganic perovskites (e.g., CsPbI_3_ and CsPbBr_3_) are more stable than organic ones, due to smaller ionic radius of Cs^+^ than those of FA^+^ and MA^+^ cations. Many works on PSCs with inorganic perovskites as light absorber have been reported. Tan et al. [31] incorporated Cs^+^ into MA/FA hybrid perovskite to improve the photostability of solar cells. Luo et al. [32] prepared a CsPbI_3_ HTM-based PSC under fully open-air conditions with a PCE of 4.13%. Kulbak et al. [33] reported CsPbBr_3_ PSCs with different HTMs and achieved a highest PCE of 6.2%. Sutton et al. [34] demonstrated a CsPbI_2_Br-based inorganic mixed halide PSC with an efficiency up to 9.8% and high ambient stability. Both Chen’s group and Liu’s group proposed a kind of carbon-based CsPbBr_3_ all-inorganic PSCs and achieved optimal efficiencies of 5.0% [35] and 6.7% [36], respectively. All these PSCs using inorganic perovskite have demonstrated a relatively enhanced stability. On the other hand, p-type semiconductor CuPc, small molecular HTMs with planar configuration, is preferable in fabricating stable and efficient traditional organic PSCs [37–39]. It owns properties of low cost, ease of synthesis, low band gap, high hole mobility of 10^−3^–10^−2^ cm^2^ V^−1^ S^−1^ (as compared with 4 × 10^−5^ cm^2^ V^−1^ S^−1^ for spiro-OMeTAD) [40], good stability (starting degradation above 500 °C in air), and long exciton diffusion length (*L*_ex_ ranging from 8 to 68 nm) [41–43]. Nonetheless, CuPc is never reported as HTM in inorganic perovskite photovoltaic devices. Besides, novel counter electrodes including Al [44], Ni [45], and carbon [46–48] have been explored in PSCs recently. Among them, carbon is thought to be the most promising for the electrode material because carbon is cheap, stable, inert to ion migration originating from perovskite and metal electrodes, inherently water-resistant, and therefore advantageous for good stability. The emergence of carbon counter electrode-based PSCs greatly lowers the cost and simplifies the procedures, rolling forward the development and commercialization of PSCs [49].

In this work, CuPc were introduced as HTM in carbon counter electrode-based CsPbBr_3_ inorganic PSCs. For comparison, HTM-free PSCs were also made as the control devices. The optimal CuPc-based device performance with an efficiency of 6.21% has been achieved, 63% higher than the HTM-free device. Systematic characterization and analysis were performed to reveal the underlying mechanism of the improvement originated from the CuPc HTM layer. Our results suggest that introducing CuPc between the perovskite layer and carbon electrode provides a simple and effective route to facilitate charge transfer and suppress charge recombination in PSCs. More importantly, our devices exhibit an outstanding durability and a promising thermal stability, compared with the HTM-free CsPbBr_3_ devices and traditional MAPbI_3_ devices.

## Experimental Section

### Synthesis of Carbon Paste

One gram polyvinyl acetate (PVAc) and 0.5 g hydroxypropyl cellulose were dissolved in 60 mL ethyl acetate. PVAc acted as the binder in the carbon film, and hydroxypropyl cellulose was used to adjust the viscosity of the carbon paste. 20 mL of the mixed ethyl acetate solution was blended with 2 g 40-nm graphite powder, 1 g 10-μm flake graphite, 1 g 40-nm carbon black, and 0.5 g 50-nm ZrO_2_ powder. The ZrO_2_ particles were introduced to enhance the scratch resistance performance of the carbon film [50, 51]. After vigorously milling for 2 h in an electro-mill (QM-QX0.4, Instrument Factory of Nanjing University), the printable carbon paste was ready.

### Device Fabrication

Perovskite thin film and solar cells were fabricated on fluorine-doped tin oxide (FTO)-glass substrate with the sheet resistance of 14 Ω sq^−1^. Diluted hydrochloric acid (2 mol L^−1^) and zinc powder were used to pattern the fluorine-doped tin oxide substrates. After ultrasonically cleaned by acetone, ethanol, and deionized (DI) water, the FTO substrates were treated under oxygen plasma for 30 min to remove the last traces of organic residues. A thin layer of compact anatase TiO_2_ with 50 nm in thickness was deposited by spin-coating a mildly acidic solution of titanium isopropoxide in ethanol at 5000 rpm for 60 s and consequently annealed at 500 °C for 30 min. After cooling down to room temperature, the mesoporous TiO_2_ scaffold (particle size 20 nm) was formed by spin-coating TiO_2_ paste (DSL. 18NR-T, 20 nm, Dyesol, Australia) diluted in ethanol (2:7 weight ratio) at 5000 rpm for 60 s and consequently heating at 500 °C for 30 min. The CsPbBr_3_ perovskite layer was prepared by a sequential method. 1.47 g PbBr_2_ was dissolved in 4 mL *N*,*N*-dimethylformamide (DMF) and heated at 80 °C for 12 h under magnetic stirring. The prepared mesoporous TiO_2_ films were preheated to ~ 80 °C and then infiltrated with the PbBr_2_ precursor solution by spin-coating at 2000 rpm for 45 s and dried at 80 °C for 30 min immediately. Sequentially, the PbBr_2_ films were immersed in a methanol solution of 0.07 M CsBr for 15 min. After rinsed by 2-propanol and dried in air, the samples were heated to 250 °C for 5 min on a hotplate to form a uniform layer of CsPbBr_3_. CuPc was deposited on the perovskite film by vacuum evaporation (< 1 × 10^−3^ Pa) using quartz crystal monitor to determine the thickness and deposition rate. The deposition of carbon CE was conducted by doctor blade method and dried at 80 °C for 15 min. All these procedures were carried out on naturally ambient atmosphere.

### Characterization

The morphology of the perovskite surface and cross-sectional structure of the solar cells was observed by the field emission scanning electron microscopy (FESEM, JSM-7600F, JEOL). The formation of CsPbBr_3_ perovskite absorber layer has been further confirmed by X-ray diffraction (XRD) analysis (PANalytical PW3040/60) with Cu Kα radiation (*λ* = 1.5406 Å) from 10° to 50°. An X-ray photoelectron spectrometer (XPS, Axis Ultra DLD, Shimadzu) equipped with a monochromatic Al Kα source (1486.6 eV) was employed to determine the surface chemical composition of CsPbBr_3_ perovskite film. The Raman spectra of the CuPc film on glass substrate were performed by a Raman spectrometer (LabRAM HR800, Horiba JobinYvon) with a 532 nm laser source. All the XPS spectra were obtained in the constant pass energy mode, where the pass energy of the analyzer was set at 20 eV. Here the binding energy of the C 1*s* peak (285 eV) arising from adventitious carbon was used for the energy calibration. UV–Vis spectrophotometer (UV 2600, Shimadzu) was utilized to obtain the absorption spectra of CsPbBr_3_ and CsPbBr_3_/CuPc films. The steady-state photoluminescence measurements were taken using a spectrometer (LabRAM HR800, Horiba JobinYvon) under an excitation laser with a wavelength of 325 nm. The time-resolved photoluminescence decay transients were measured at 525 nm using excitation with a 478-nm light pulse from a HORIBA Scientific DeltaPro fluorimeter. Current density–voltage (*J*–*V*) curves were recorded under AM 1.5, 100 mW cm^−2^ simulated sunlight (Oriel 94043A, Newport Corporation, Irvine, CA, USA) with an electrochemical station (Autolab PGSTAT302 N, Metrohm Autolab, Utrecht, The Netherlands), previously calibrated with an NREL-calibrated Si solar cell. The measurements were taken with a black metal mask with a circular aperture (0.071 cm^2^) smaller than the active area of the square solar cell (1.5 × 1.5 cm^2^). The incident photon to current conversion efficiency (IPCE) was performed employing a xenon lamp coupled with a monochromator (TLS1509, Zolix) controlled by a computer.

## Results and Discussion

**Fig. 1:**
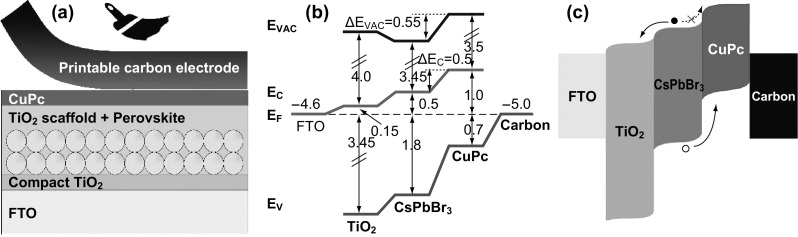
**a** Schematic cross-sectional view of the CuPc-based CsPbBr_3_ PSC with a printable low-temperature carbon electrode. **b** Schematic energy-level alignment at interfaces. *E*_VAC_ is the vacuum energy, *E*_C_ is the conduction band edges, *E*_F_ is the Fermi levels, and *E*_V_ is the valence band edges. **c** Schematic illustration of proposed band bending at interfaces

Figure 1a shows the schematic cross-sectional view of the CuPc-based CsPbBr_3_ PSC. The cell consists of functional layers of FTO/compact TiO_2_/mesoporous TiO_2_/inorganic perovskite CsPbBr_3_/CuPc/carbon. Figure 1b displays the schematic energy-level alignment of the PSCs device. The TiO_2_ compact layer is used as the electron-collecting layer, and the mesoporous TiO_2_ layer is used as the scaffold for light-sensitive absorption material. According to previous study, the *E*_C_, *E*_F_, and *E*_V_ of TiO_2_ are 4.0, 4.15, and 7.6 eV, respectively [52]. The counter electrode is printed by a low-temperature printable carbon paste. Compared to the traditional organometal CH_3_NH_3_PbI_3_ perovskite material, CsPbBr_3_ owns a wider band gap of 2.3 eV, a work function of 3.95 eV, and a valence band energy of 5.75 eV [53]. CuPc is a typical organic small molecular photoelectric semiconductor material with the corresponding molecular structure as shown in Fig. S1. The highest occupied molecular orbital (HOMO) and the lowest unoccupied molecular orbital (LUMO) levels of CuPc are ascribed to − 5.2 and − 3.5 eV, respectively [54]. What is more, the gap between *E*_F_ and *E*_V_ is reported to be 0.7 eV [55]. It can be found that the device exhibits a smooth energy-level transition by using CuPc as the HTM. The proposed band bending at interfaces is illustrated in Fig. 1c. Under illumination, free charge carriers generated in the CsPbBr_3_ layer can be extracted by transferring electrons (filled circle) and holes (open circle) to TiO_2_ and CuPc, since the energy-level alignments are appropriate. Besides, the conduction band offset between the CsPbBr_3_ layer and CuPc layer (0.5 eV) provides an energy barrier that prevents photogenerated electrons from flowing to the CuPc layer, whereas the valence band offset provides an additional driving force for the flow of photogenerated holes to the CuPc layer. The insertion of the CuPc layer not only prevents electron flow from CsPbBr_3_ to the anode but may also reduce surface recombination of photogenerated electrons and holes at the CsPbBr_3_/carbon interface [46, 56]. The final collection of the holes is extracted by carbon through the CuPc/carbon interface from the HOMO of CuPc (− 5.2 eV) to carbon (− 5.0 eV), while electrons are collected by FTO (− 4.6 eV) [47, 48].

**Fig. 2:**
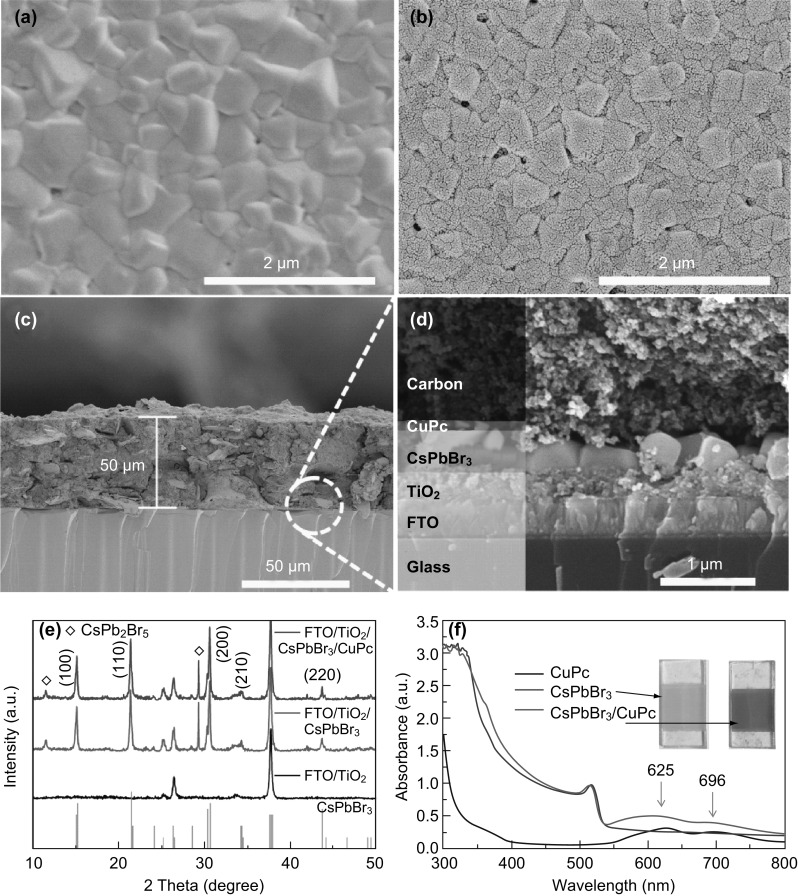
SEM images of **a** top view of the CsPbBr_3_ perovskite film and **b** top view of the CsPbBr_3_ perovskite film deposited with CuPc. **c** Cross-sectional SEM image of the whole device. **d** Close-up of the structure under higher magnification. **e** XRD spectra of the FTO/TiO_2_, FTO/TiO_2_/CsPbBr_3_, and FTO/TiO_2_/CsPbBr_3_/CuPc. **f** UV–Vis absorbance spectra of the CuPc, FTO/TiO_2_/CsPbBr_3_, and FTO/TiO_2_/CsPbBr_3_/CuPc

The film quality of the CsPbBr_3_ and the surface morphology of the CsPbBr_3_ film with CuPc on the top, as well as the cross-sectional view of the whole device, are measured by high-resolution SEM, as depicted in Fig. 2a–d. The as-formed CsPbBr_3_ film possesses the characteristic of well surface coverage on the substrate. Relatively uniform grain size ranging from 100 to 1000 nm can be derived from Fig. 2a. However, striking different top-view morphology is revealed by depositing CuPc on the surface of the perovskite grains (Fig. 2b). The higher-magnification image derived from Fig. S2 demonstrates a nanorod-like morphology of the CuPc aggregated by layered deposition. Decorated with the CuPc film, the perovskite grains become sea cucumber-like. The molecular interactions in the CuPc nanorods are enhanced due to the strong *π*–*π* stacking between the layered CuPc molecular, which favors the formation of high carrier mobility to some extent. [57] Moreover, depositing thin CuPc can also compensate some defects on the surface of the CsPbBr_3_ as well as induce a large interfacial area of the CuPc, which is conductive to a good contact with the counter electrode, correspondingly favoring the hole transported from the CuPc to the carbon. The cross-sectional SEM images of the whole device shown in Fig. 2c, d demonstrate a well-defined layer-by-layer structure with sharp interfaces. The thickness of the mp-TiO_2_, perovskite capping layer, and carbon layers is determined as 600 nm, 500 nm, and 50 μm, respectively. The CuPc layer is too thin to be identified in this scale. The line-scan analysis of EDX map is further conducted to investigate the distribution of components in the solar cell, as shown in Fig. S3. The evident peak from Cu proves the existence of CuPc between the interface of CsPbBr_3_ and carbon. According to the result of four-point probe resistivity measurement, the carbon electrode shows a sheet resistance of around 70 Ω sq^−1^ and exhibits a good electrical conductivity. Figure 2e shows the XRD patterns of the FTO/TiO_2_ (black curve), FTO/TiO_2_/CsPbBr_3_ (red curve), and FTO/TiO_2_/CsPbBr_3_/CuPc (blue curve) films. Obvious diffraction peaks at 15.1°, 21.4°, 30.6°, 34.3°, and 43.7° are consistent with the planes of (100), (110), (200), (210), and (220) of CsPbBr_3_, respectively [35]. Impure peaks at 11.6° and 29.3° (marked by rhombuses) may attribute to (002) and (220) planes of by-product CsPb_2_Br_5_, which is hard to eliminate when to obtain CsPbBr_3_. The occurrence of CsPb_2_Br_5_ in the final product can be attributed to the metastable state in the cubic phase, non-stoichiometric material transfer, or structural rearrangement [58, 59]. The generation of secondary-phase CsPb_2_Br_5_ in the product can be ascribed to the following process:

1 $${\text{PbBr}}_{2} + {\text{CsPbBr}}_{3} \to {\text{CsPb}}_{2} {\text{Br}}_{5}$$

The excess PbBr_2_ or the poor solubility of CsBr in methyl alcohol could facilitate the transformation at a low temperature [60]. CsPb_2_Br_5_ crystal is reported to exhibit an inactive photoluminescence behavior and a large indirect band gap of approximately 3.1 eV [61], which are unfavorable in the application of photovoltaic device and need to be eliminated by future process optimization. After coated by CuPc, the XRD patterns of the film show negligible changes, mainly due to the amorphous state of the CuPc [42]. In the Raman spectra (Fig. S4), the peak at 680.2 cm^−1^ is ascribed to the breathing vibration band of phthalocyanine ring, the peak at 1140.5 cm^−1^ is ascribed to the breathing vibration band of benzene ring, and the peaks at 1137.5, 1452.0, and 1526.5 cm^−1^ are attributed to the stretching vibration band of C–C, C–N, and C=C bond, respectively [62]. UV–Vis absorbance spectra of the CuPc, FTO/TiO_2_/CsPbBr_3_, and FTO/TiO_2_/CsPbBr_3_/CuPc are also demonstrated in Fig. 2f. The CsPbBr_3_ film strongly absorbs light with the wavelength between 300 and 540 nm, owning to the relatively wide band gap (2.3 eV) as shown in Fig. S5. Pristine CuPc demonstrates a wide spectral ranging from 500 to 800 nm, and peaks at 625 and 696 nm, which are ascribed to the Q-band of CuPc. The peak at 625 nm is the absorbance peak of the CuPc dimer, and the peak at 696 nm comes from the CuPc monomer [63, 64]. In the presence of CuPc, an enhancement in absorption is observed, especially in the region of 537–800 nm. Correspondingly, the color of the films changes from golden yellow to light green, as shown in the inset of Fig. 2f.

**Fig. 3:**
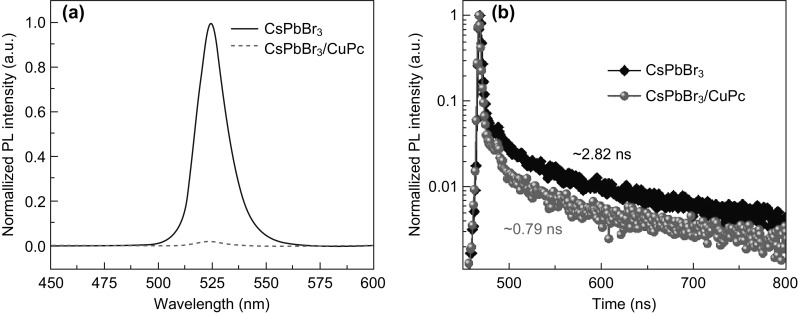
Steady-state **a** and time-resolved **b** PL spectra of the CsPbBr_3_ and CsPbBr_3_/CuPc films

Steady-state PL and time-resolved photoluminescence (TRPL) are judiciously employed for the CsPbBr_3_ film and the CsPbBr_3_ film coated by CuPc, which are deposited on the quartz glasses. As shown in Fig. 3a, both samples under the same laser pulse energy exhibit PL emission peaks at 525 nm arising from the CsPbBr_3_ perovskite layer. Significant quenching effect is observed when the perovskite layer interfaces with the CuPc layer, indicating that CuPc is effective in hole extraction. This owes to the high mobility and high interfacial film quality together with intimate contact with the CsPbBr_3_ film formed by excellent *π*–*π* stacking. In order to evaluate the hole-extraction rate and bimolecular recombination process of the free electrons and holes, TRPL decay is further performed via monitoring the peak emission at 525 nm. The results are shown in Fig. 3b. The excitation impinges on the sample from the glass side with a pulsed laser at 478 nm. By biexponential fitting of the dynamic TRPL curve, the pure CsPbBr_3_ perovskite film exhibits a carrier lifetime of 2.82 ns, whereas the addition of nanorod-like CuPc accelerates the PL decay with an observed carrier lifetime of 0.79 ns. Here the carrier lifetime in the perovskite film describes various radiative and non-radiative loss channels responsible for photoexcited carrier recombination [65]. The smaller lifetime induced by the CuPc nanorods quenching indicates a fast hole-diffusion process, a reduced trap-assisted recombination, and an efficient hole-extraction capability [40].

**Fig. 4:**
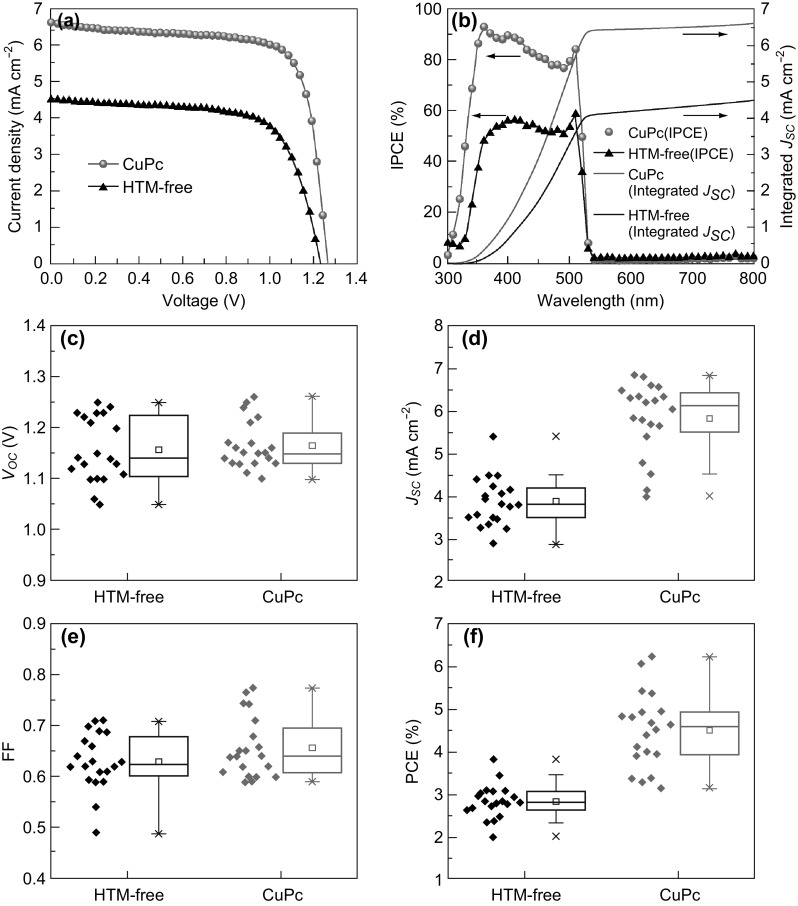
**a** Best performed current density–voltage (*J*–*V*) curves of typical small-area (0.071 cm^2^) carbon-based CsPbBr_3_ PSCs with CuPc as HTM or without HTM measured under 100 mW cm^−2^ photon flux (AM 1.5G), respectively. **b** IPCE spectra and corresponding integrated photocurrents. Box charts exhibiting the statistical features of **c** *J*_SC_, **d** *V*_OC_, **e** FF, and **f** PCE of the carbon-based CsPbBr_3_ PSCs with CuPc as HTM or without HTM

The photovoltaic performances of the devices with 60-nm CuPc as HTM or without any HTM were characterized by *J*–*V* measurements under simulated AM 1.5G solar irradiation at 100 mW cm^−2^ in the air (Fig. 4a). The results are summarized in Table 1. The optimized device with CuPc as HTM shows a short-circuit current density (*J*_SC_) of 6.62 mA cm^−2^, an open-circuit voltage (*V*_OC_) of 1.26 V, a fill factor (FF) of 0.74, and a champion PCE of 6.21%, showing 63% enhancement than the HTM-free device (3.8%). The corresponding IPCE spectra are displayed in Fig. 4b. The IPCE starts to increase at 540 nm, consistent with the UV–Vis spectrum of CsPbBr_3_. After applying CuPc as the HTM, the IPCE shows noticeable improvement in the region between 300 and 540 nm due to more effective charge collection and extraction. Note that the IPCE curve also exhibits a blue-shift character, leading to an effective use for the near-ultraviolet light. Integrating the overlap of the IPCE spectra of the CuPc-based and HTM-free devices yields the current density of 6.58 and 4.48 mA cm^−2^, respectively, in good agreement with the experimentally obtained *J*_SC_. Figure 4c–f is box charts exhibiting the statistical features (*J*_SC_, *V*_OC_, FF, and PCE) of the carbon-based CsPbBr_3_ PSCs with CuPc as HTM and those without HTM. It is obvious that the PCE is enhanced after introducing CuPc, mainly ascribing to the improved *J*_SC_. Moreover, the thickness of the CuPc layer also plays a vital role in affecting the performance of the solar cells (see Fig. S7 and Table S2). If the CuPc layer is too thin, the hole transportation function of the CuPc will not work effectively; if the CuPc is too thick, the series resistance of the device will increase due to relatively low conductivity of the pristine CuPc, resulting in a poor performance. Thus, the optimized thickness of the CuPc layer is very important, and 60 nm is obtained in this study. Generally, PSCs suffer from hysteresis phenomena: *V*_OC_ and FF varied under different scan directions. As displayed in Fig. S8, despite the improvement in efficiency after introducing CuPc as HTM, our device still suffer from severe hysteresis effect: Efficiency at forward scan (3.12%) is only 54% of the efficiency at reverse scan (5.74%). Recent studies suggest that *J*–*V* hysteresis is related to the presence of defects and trap states at the perovskite/electron transport layer and/or perovskite/hole transport layer interfaces [66–69]. The combination of ion migration in the perovskite film and interfacial recombination is thought to be responsible for many of the observed hysteresis behaviors [70–72]. These phenomena may be further eliminated via interfacial modification or interface passivation [73–77].

**Table 1:** Photovoltaic performance of the TiO_2_/CsPbBr_3_/CuPc/carbon devices and TiO_2_/CsPbBr_3_/carbon devices measured under simulated AM 1.5G (100 mW cm^−2^) condition

Cell configuration	*V*_OC_ (V)	*J*_SC_ (mA cm^−2^)	FF	PCE (%)
TiO_2_/CsPbBr_3_/CuPc/carbon				
Champion	1.26	6.62	0.74	6.21
Average	1.17	5.83	0.66	4.47
TiO_2_/CsPbBr_3_/carbon				
Champion	1.23	4.50	0.69	3.80
Average	1.16	3.87	0.63	2.81

**Fig. 5:**
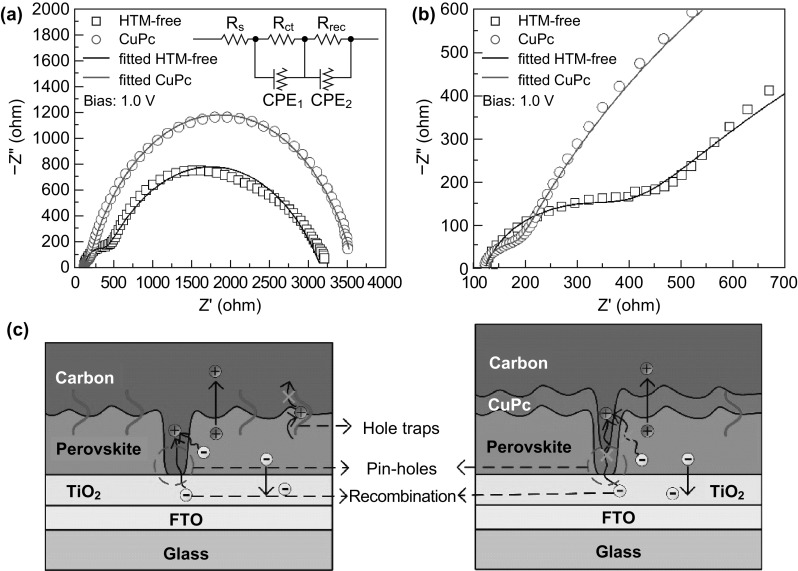
**a** Nyquist plots at bias 1.0 V of the HTM-free and CuPc-based CsPbBr_3_ PSCs in the dark. Inset: the equivalent circuit employed to fit the Nyquist plots. **b** Nyquist plots in the high frequency range. **c** Illustration depicting the function of CuPc as HTM layer in PSCs

To further evaluate the recombination in the HTM-free and CuPc-based CsPbBr_3_ PSCs, EIS was applied to track the interface charge behavior. Measurements were taken at a bias of 1.0 V in the frequency ranging from 10^7^ to 10 Hz under dark condition. Figure 5a, b shows the Nyquist plots of two devices (with CuPc or HTM-free), and an equivalent circuit (inset in Fig. 5a) is used to fit the curves. As can be found, there are two well-defined semicircles, including a small one in the high frequency range (magnified in Fig. 5b) and a large one in the low frequency range. The right semicircle in the low frequency range is mainly attributed to the recombination resistance (*R*_rec_) at the TiO_2_/perovskite interface. The small semicircle corresponding to the high-frequency part stands for the charge transfer resistance (*R*_ct_) at the perovskite/HTM or perovskite/carbon interface [78–80]. Compared with the HTM-free CsPbBr_3_ PSC, the *R*_rec_ increases from 2.87 to 3.41 kΩ after using CuPc as HTM in the device. The larger *R*_rec_ of the device with CuPc at the same forward bias voltage suggests that CuPc as the HTM is superior in preventing charge recombination. Furthermore, introducing CuPc will also lower the *R*_ct_ (from 213.0 to 45.6 Ω), indicating a more efficient charge transfer process than that occurred in the HTM-free device. All these results lead to an enhanced *J*_SC_ and thereby an improved PCE. A further explanation is that the appropriate energy level and high hole mobility of the CuPc may help to accelerate the extractions of photon-generated carriers, resulting in a larger *R*_rec_ and a smaller *R*_ct_. The favorable effect of the CuPc HTM layer on the device operation is summarized by the device models shown in Fig. 5c, similar to the planar heterojunction organic photovoltaic devices [81]. A perforation in the perovskite film (circled by dash line in red) is sketched for better illustration of the working mechanism of our devices. It does not indicate that the perovskite film is totally discontinuous since the hole is enlarged for clarity. In perovskites, bimolecular recombination is caused by the recombination of photogenerated electrons and holes, whereas monomolecular recombination is from photoexcited carriers and unintentionally trap states [82]. It is proposed that large perovskite grains with few trap states show bimolecular recombination and high device efficiency, whereas the perovskite films with trap states present monomolecular recombination and low device efficiency [3]. The carriers trapped by the trap states will lead to slow response of the photocurrent through the delay in charge transport by trapping and detrapping processes and cause losses in carrier collection which will lead to a low *J*_SC_. Hence, there are mainly two reasons for the enhanced photovoltaic performance of the CuPc-based PSCs. First, pin-holes in the perovskite layer (as shown in Fig. S9), which are hard to eliminate by technological means, will lead to the formation of defects, acting as centers to facilitate the recombination of holes and electrons. Introducing CuPc as HTM layer can build a Schottky barrier at the perovskite/carbon interfaces and suppress carrier recombination [83, 84]. Second, the CuPc HTM layer provides a smoother energy-level transition, reducing trap states and monomolecular recombination, which will benefit for high efficient solar cells [3, 85].

**Fig. 6:**
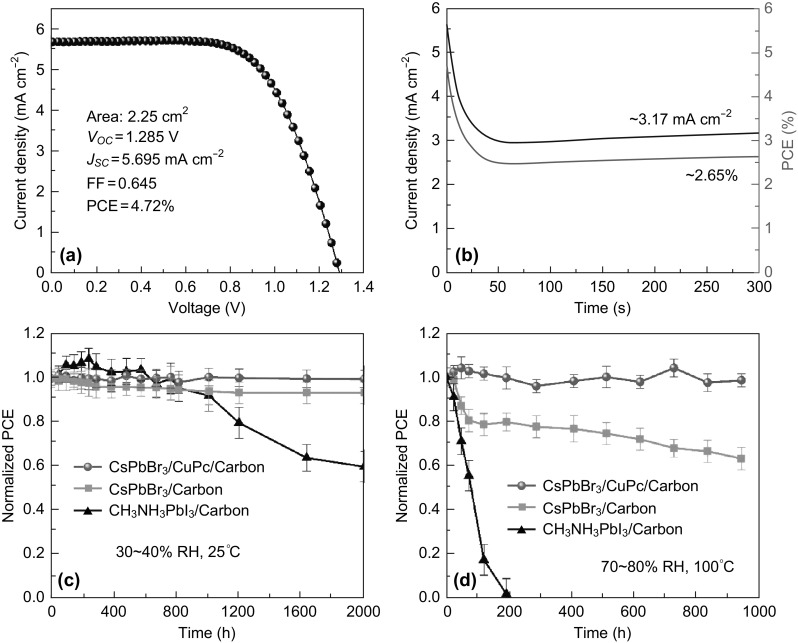
**a** *J*–*V* plot of the carbon-based perovskite solar cell with a large active area of 2.25 cm^2^. **b** Stabilized power output measurement for the large-area PSC. **c** Normalized PCE of CsPbBr_3_/CuPc/carbon-, CsPbBr_3_/carbon-, and CH_3_NH_3_PbI_3_/carbon-based PSCs versus storage time in ambient air (30–40% RH, 25 °C) without encapsulation. **d** Normalized PCE of CsPbBr_3_/CuPc/carbon-, CsPbBr_3_/carbon-, and CH_3_NH_3_PbI_3_/carbon-based PSCs versus storage time heated at high temperature (100 °C) in a high-humidity ambient environment (70–80% RH, 100 °C) without encapsulation

Moreover, CuPc-based CsPbBr_3_ PSCs with large active area (2.25 cm^2^) were also fabricated. Figure 6a shows the *J*–*V* plots of a large-area PSC under AM1.5 G standard solar light. The device shows a *V*_oc_ of 1.285 V, a *J*_sc_ of 5.695 mA cm^−2^, a FF of 0.645, reaching a PCE of 4.72%. Measuring the steady-state power output directly at a given bias is also feasible to estimate the PCE. As shown in Fig. 6b, we recorded the photocurrent density of the device held at a forward bias of 0.85 V near its maximum output power as a function of time, so as to monitor the stabilized power output under working conditions. The photocurrent density stabilizes within seconds to approximately 3.17 mA cm^−2^, yielding the stabilized power conversion efficiency around 2.65% measured after 300 s. Here, the decay of *J*sc toward the steady current is ascribed to the capture of build-up holes in surface states associated with recombination [86], similar to that occurred in traditional organic–inorganic hybrid PSCs [34, 87].

Long-term stability is a critical concern for practical applications of PSCs. Figure 6c presents the room-temperature stability test of the CuPc-based CsPbBr_3_ PSCs in comparison with the HTM-free CsPbBr_3_ devices and the classical CH_3_NH_3_PbI_3_/carbon devices. The devices without encapsulation were stored in dark with a humidity of 30–40% RH. Both the CuPc-based CsPbBr_3_ PSCs and HTM-free CsPbBr_3_ PSCs exhibit excellent stability beyond 2000 h, while the organic ones start degrading at 800 h. Thermal stability of the devices was further evaluated in a harsh environment (the humidity of 70–80% RH and the temperature of 100 °C), as shown in Fig. 6d. Obviously, the performance of CH_3_NH_3_PbI_3_/carbon devices decays rapidly, since the high humidity and high storing temperature accelerate the degradation of CH_3_NH_3_PbI_3_ perovskite light absorber. The HTM-free CsPbBr_3_ devices also show a PCE loss of 37% after 944 h, similar to the previous research [35]. However, the CuPc-based CsPbBr_3_ devices show an outstanding thermal stability (without evident decay) during the whole testing period. The organic CH_3_NH_3_^+^ cation is more vulnerable to moisture and has higher volatility than the inorganic Cs^+^ cation, leading to rapid degradation of the CH_3_NH_3_PbI_3_ devices under relatively high RH and temperature environment [35]. The introduction of the CuPc film and carbon film, which can act as shields to prevent the deliquescing of the underlying perovskite layer, obtains the best hydrophobicity and thus results in the best stability of CuPc-based CsPbBr_3_ PSCs.

## Conclusion

In summary, cost-effective p-type material CuPc was introduced as HTM layer in the carbon-based CsPbBr_3_ inorganic PSCs. The deposited CuPc layer exhibits a nanorods morphology and an intimate contact with the perovskite layer, preventing direct contact between the perovskite layer and carbon electrode. The CuPc layer can effectively extract the photon-generated carriers and accelerate the hole-diffusion process, obtaining a decent PCE (6.21%) with high reproducibility. Compared with HTM-free CsPbBr_3_/carbon devices, the enhanced PCE may be ascribed to a more efficient charge transfer and a more suppressed charge recombination. Moreover, the newly developed devices demonstrate a dramatically enhanced durability under ambient atmosphere and a promising thermal stability in relatively harsh condition. The enhanced PCE and excellent stability of our devices offer a new device designing strategy and promise a reality of commercial application for PSCs with cost-effective, mass manufacturing solar technology that is compatible with current large-scale printing infrastructure.

## Electronic supplementary material

Below is the link to the electronic supplementary material.

Supplementary material 1 (PDF 895 kb)
