# Supplementary material for: Efficient Carbon-Based CsPbBr3 Inorganic Perovskite Solar Cells by Using Cu-Phthalocyanine as Hole Transport Material
Source: Nanomicro Lett. 2018 Jan 16;10(2):34. doi: 10.1007/s40820-018-0187-3 (PMC6199080; doi:10.1007/s40820-018-0187-3)
Supplement: Supplementary file 1 — Supplementary material 1 (PDF 895 kb) [file 40820_2018_187_MOESM1_ESM.pdf]

Supporting Information for

Efficient Carbon Based $CsPbBr_3$ Inorganic Perovskite Solar Cells by Using Cu-Phthalocyanine as Hole Transport Material

Zhiyong Liu[1], Bo Sun[1], Xingyue Liu[1], Jinghui Han[1], Haibo Ye[1], Tielin Shi[1], Zirong Tang[1], Guanglan Liao[1, 2, *]

[1]State Key Laboratory of Digital Manufacturing Equipment and Technology, Huazhong University of Science and Technology, Wuhan 430074, People's Republic of China

[2]Flexible Electronics Research Center, Huazhong University of Science and Technology, Wuhan 430074, People's Republic of China

*Corresponding author. E-mail: guanglan.liao@hust.edu.cn

Figures and Tables

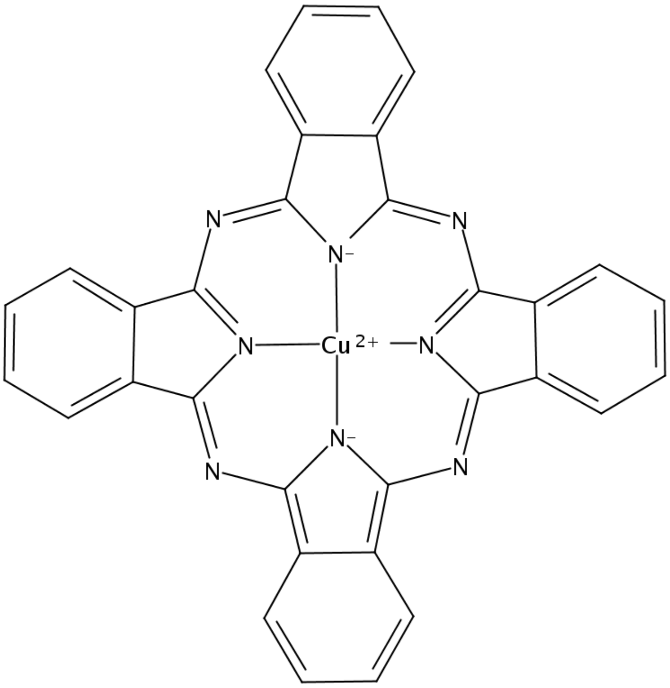

Fig. S1 Molecular structure of copper phthalocyanine (CuPc)

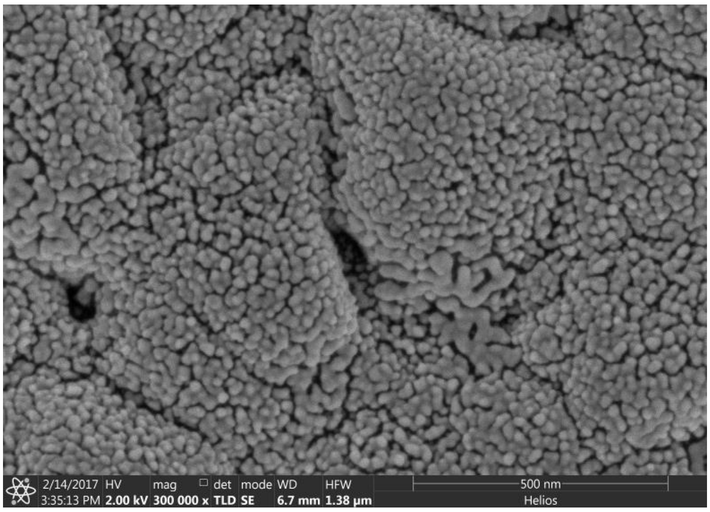

Fig. S2 SEM image of top-view of the $CsPbBr_3$ layer deposited with CuPc under a big magnification

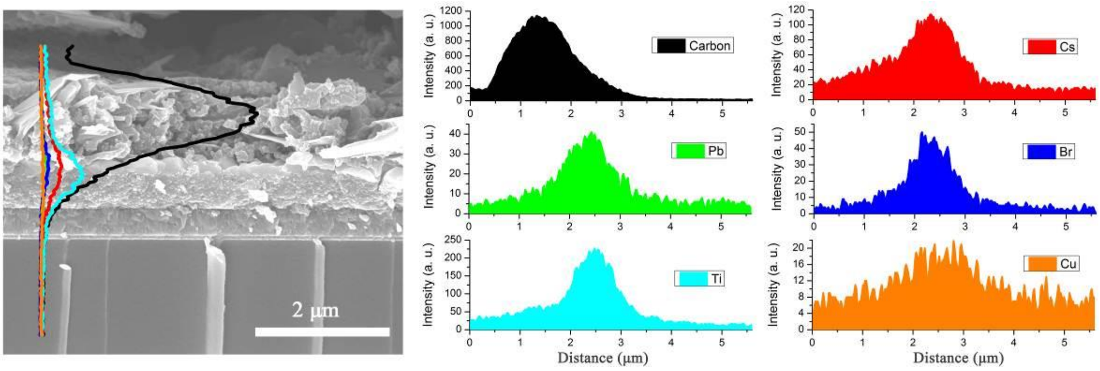

Fig. S3 Distribution of components in the carbon based $CsPbBr_3$ PSC obtained by the line-scan analysis of EDX map

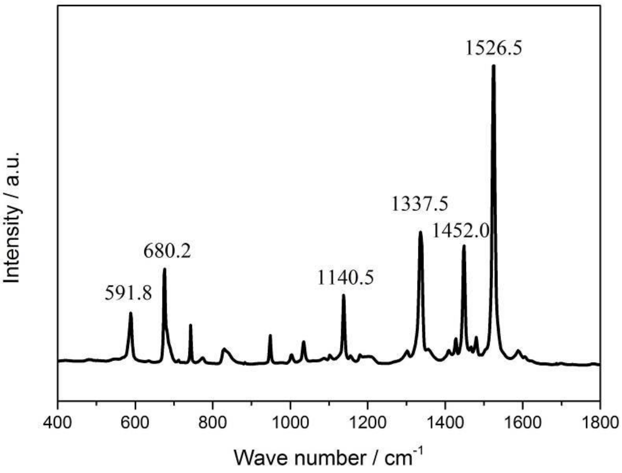

Fig. S4 Raman spectra of the CuPc film deposited on glass substrate

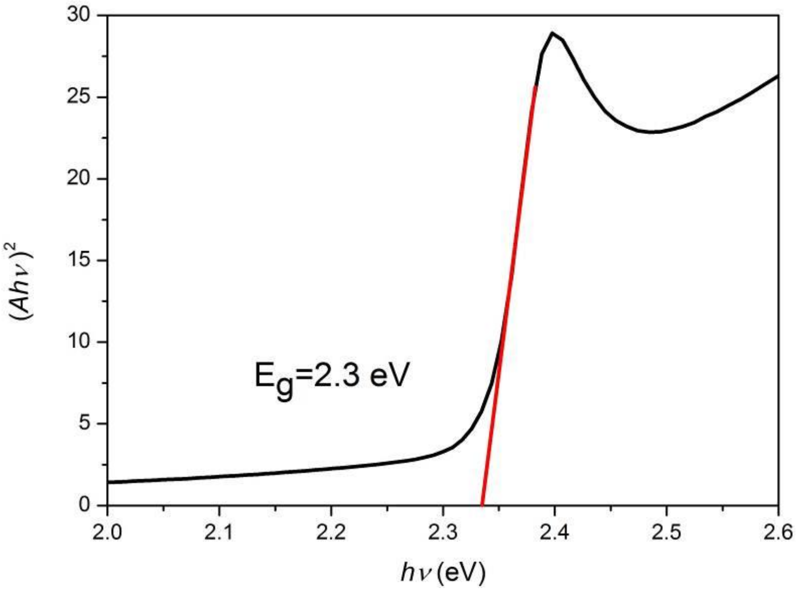

Fig. S5 $(Ahv)^2$ *vs.* energy (*hv*) curve of $CsPbBr_3$ film calculated from the absorbance spectrum. The optical band gap of $CsPbBr_3$ is measured to be ~2.3 eV

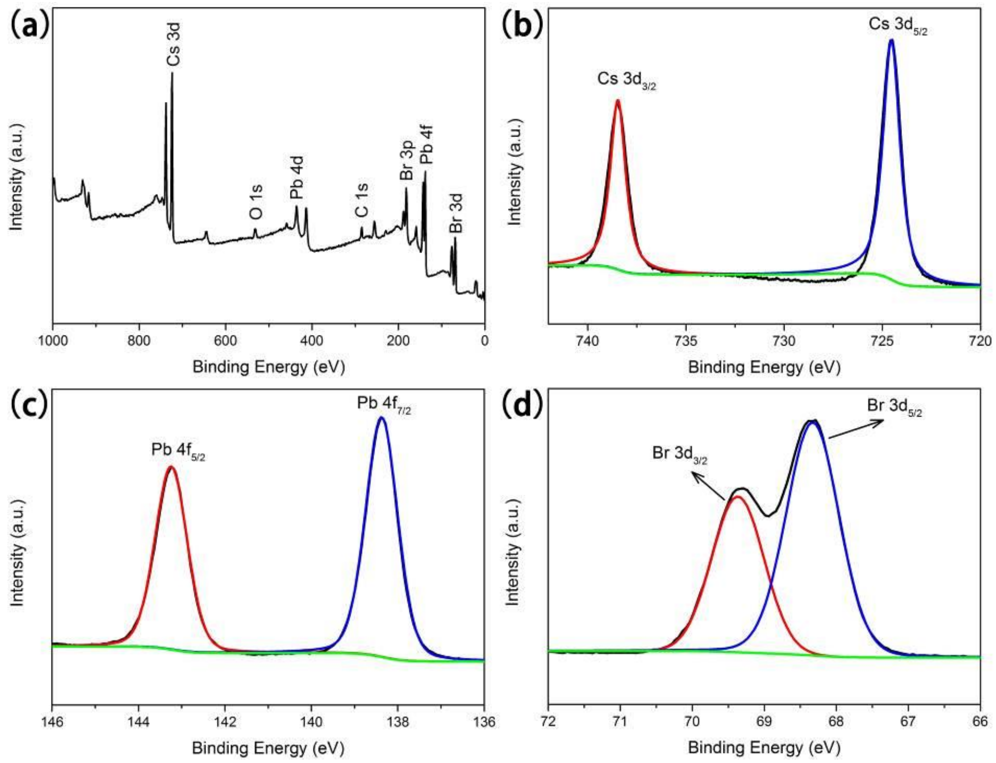

Fig. S6 XPS characterizations of $CsPbBr_3$ film. **a** Survey XPS spectrum of $CsPbBr_3$ film. Corresponding high-resolution XPS spectra of the **b** Cs 3d, **c** Pb 4f and **d** Br 3d regions

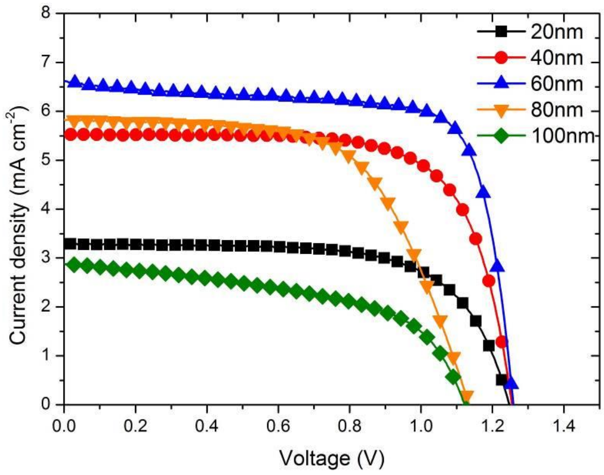

Fig. S7 *J*-*V* curves for the PSCs with different CuPc thickness

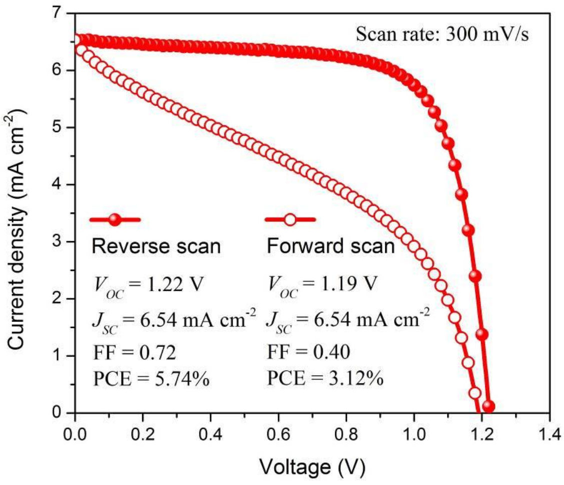

Fig. S8 *J*-*V* curves of the carbon based $CsPbBr_3$ PSC under both forward and reverse scans. The scan rate is 300 mV s^{-1}

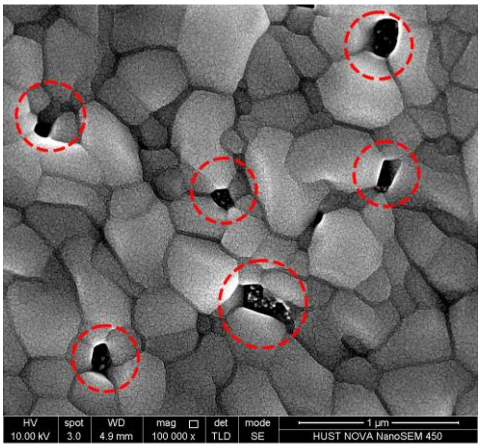

Fig. S9 Pin-holes on the $CsPbBr_3$ perovskite surface

Table S1 XPS binding energies and atomic ratios of Cs, Pb, and Br elements measured from the inorganic perovskite $CsPbBr_3$ layer

	**Cs 3d5/2**	**Cs 3d3/2**	**Pb 4f7/2**	**Pb 4f5/2**	**Br 3d5/2**	**Br 3d3/2**
**Binding energy (eV)**	724.5	738.4	138.4	143.2	68.3	69.3
**Atomic ratio (%)**	21.4		21.4		57.2	

Table S2 Parameters of the PSCs with different CuPc thickness

**CuPc thickness (nm)**	V_{OC} **(V)**	J_{SC} **(mA cm^{-2})**	**FF**	**PCE (%)**
20	1.25	3.27	0.67	2.74
40	1.25	5.53	0.71	4.93
60	1.26	6.62	0.74	6.21
80	1.13	5.83	0.62	4.07
100	1.12	2.88	0.53	1.72
